# *In Vivo* Efficacy and Metabolism of the Antimalarial Cycleanine and Improved *In Vitro* Antiplasmodial Activity of Semisynthetic Analogues

**DOI:** 10.1128/AAC.01995-20

**Published:** 2021-01-20

**Authors:** Fidelia Ijeoma Uche, Xiaozhen Guo, Jude Okokon, Imran Ullah, Paul Horrocks, Joshua Boateng, Chenggang Huang, Wen-Wu Li

**Affiliations:** a School of Pharmacy and Bioengineering, Keele University, Stoke-on-Trent, United Kingdom; b Shanghai Institute of Materia Medica, Chinese Academy of Science, Shanghai, China; c Department of Pharmacology and Toxicology, Faculty of Pharmacy, University of Uyo, Uyo, Nigeria; d School of Medicine, Keele University, Stoke-on-Trent, United Kingdom; e School of Science, University of Greenwich, United Kingdom

**Keywords:** malaria, *Plasmodium falciparum*, *Plasmodium berghei*, bisbenzylisoquinoline alkaloids, cycleanine, metabolism, *in vivo* activity, antimalarial agents, drug metabolism

## Abstract

Bisbenzylisoquinoline (BBIQ) alkaloids are a diverse group of natural products that demonstrate a range of biological activities. In this study, the *in vitro* antiplasmodial activity of three BBIQ alkaloids (cycleanine [compound 1], isochondodendrine [compound 2], and 2′-norcocsuline [compound 3]) isolated from the Triclisia subcordata Oliv. medicinal plant traditionally used for the treatment of malaria in Nigeria are studied alongside two semisynthetic analogues (compounds 4 and 5) of cycleanine. The antiproliferative effects against a chloroquine-resistant Plasmodium falciparum strain were determined using a SYBR green 1 fluorescence assay. The *in vivo* antimalarial activity of cycleanine is then investigated in suppressive, prophylactic, and curative murine malaria models after infection with a chloroquine-sensitive Plasmodium berghei strain. BBIQ alkaloids (compounds 1 to 5) exerted *in vitro* antiplasmodial activities with 50% inhibitory concentration (IC_50_) at low micromolar concentrations and the two semisynthetic cycleanine analogues showed an improved potency and selectivity compared to those of cycleanine. At oral doses of 25 and 50 mg/kg body weight of infected mice, cycleanine suppressed the levels of parasitemia and increased mean survival times significantly compared to those of the control groups. The metabolites and metabolic pathways of cycleanine were also studied using high-performance liquid chromatography–electrospray ionization–tandem mass spectrometry. Twelve novel metabolites were detected in rats after intragastric administration of cycleanine. The metabolic pathways of cycleanine were demonstrated to involve hydroxylation, dehydrogenation, and demethylation. Overall, these *in vitro* and *in vivo* results provide a basis for the future evaluation of cycleanine and its analogues as leads for further development.

## Abstract (precis)

Bisbenzylisoquinoline (BBIQ) alkaloids are a diverse group of natural products that demonstrate a range of biological activities. In this study, the *in vitro* antiplasmodial activity of three BBIQ alkaloids (cycleanine [compound 1], isochondodendrine [compound 2], and 2′-norcocsuline [compound 3]) isolated from the Triclisia subcordata Oliv. medicinal plant traditionally used for the treatment of malaria in Nigeria are studied alongside two semisynthetic analogues (compounds 4 and 5) of cycleanine.

## TEXT

In 2018, the World Health Organization (WHO) report estimated a global burden of 228 million cases accounting for 405,000 deaths (1). The majority of this burden fell on the WHO Africa Region, where malaria, particularly that caused by the most virulent etiological agent Plasmodium falciparum, exerts an immense economic impact. While malaria cases and mortality figures continue to fall (1, 2), the development and spread of resistance to available chemotherapeutic agents poses a significant threat to malaria treatment and management (3). Natural products of plant origin have traditionally provided good sources for discovery of drug leads or novel compounds in modern drug research (4, 5). For example, artemisinin isolated from Artemisia annua, sweet wormwood, a traditional Chinese medicine, together with a series of its semisynthetic derivatives, has become the first-line therapy for P. falciparum malaria (6, 7). However, due to the development of artemisinin drug resistance (8), novel therapies are still urgently needed.

**FIG 1:**
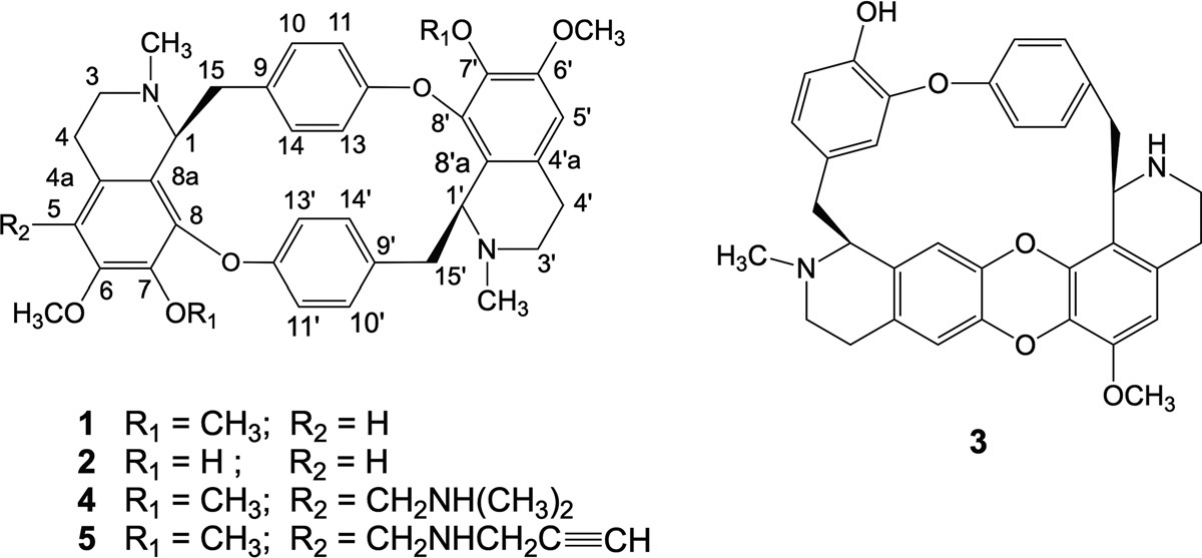
Chemical structure of bisbenzylisoquinoline (BBIQ) alkaloids. Cycleanine (compound 1), isochondodendrine (compound 2), and 2′-norcocsuline (compound 3) from T. subcordata and two novel semisynthetic analogues (compounds 4 and 5) of cycleanine.

Bisbenzylisoquinoline (BBIQ) alkaloids are a diverse group of natural products consisting of two benzylisoquinoline groups (9). BBIQ alkaloids are primarily found in the Berberidaceae, Lauraceae, Menispermaceae, and Ranunculaceae plant families. These alkaloids possess a variety of biological activities, which include antimalarial activities (9, 10). For example, BBIQ alkaloids isolated and identified from Triclisia species of the *Menispermaceae* family have antiproliferative activities (10). In Nigeria, the root of Triclisia subcordata Oliv. is traditionally used for the treatment of a range of diseases, including malaria (11, 12). The bioactive components of T. subcordata are the BBIQ alkaloids cycleanine (compound 1), isochondodendrine (compound 2) and 2′-norcocsuline (compound 3) (Fig. 1) and have previously been isolated and characterized by our group (13, 14). We have also produced synthetic analogues of cycleanine (compounds 4 and 5) (Fig. 1) (15). The three naturally occurring BBIQ alkaloids, cycleanine (16–18), isochondodendrine (18, 19), and 2′-norcocsuline (16, 20) have been reported to possess antiplasmodial effects against chloroquine-sensitive and chloroquine-resistant P. falciparum strains. Despite the promising *in vitro* biological activity of these natural BBIQ alkaloids, the *in vivo* antimalarial activity of BBIQ alkaloids has not been evaluated, nor has their potential *in vivo* metabolism. Here, we assess the *in vivo* antimalarial activity and metabolism of cycleanine (compound 1). The effect of cycleanine analogues (compounds 4 and 5) on antiplasmodial potency and selectivity is also investigated.

This study sets out an evaluation of the *in vitro* antimalarial activities of BBIQ alkaloid compounds 1 to 3 compared to those of two semisynthetic BBIQ alkaloids (compounds 4 and 5) derived by a modification of cycleanine at the C-5 position by introducing additional secondary or tertiary amine moieties in an attempt to increase potential solubility and potency (15). The most abundant BBIQ alkaloid in *T. subcordata* extract is cycleanine; this was therefore used to establish *in vivo* antimalarial activity in a murine malaria model. In addition, the metabolites and metabolic pathways of cycleanine were analyzed after intragastric administration in rats to help understand how cycleanine is eliminated *in vivo* to guide future optimization of cycleanine for antimalarial development.

## RESULTS

### The semisynthetic derivatives of cycleanine have improved *in vitro* antiplasmodial activity and selectivity.

The *in vitro* antiplasmodial activities of the five BBIQ alkaloids (compounds 1 to 5), as well as that of a chloroquine control, were determined against intraerythrocytic stages of the P. falciparum Dd2 chloroquine-resistant strain using a malaria SYBR green I fluorescence assay. These data are provided in Table 1 (see Fig. S1 in the supplemental material) as 50% inhibitory concentration (IC_50_) values (mean ± standard deviation [SD] for *n* = 3 independent biological repeats). While the data for chloroquine in Dd2 are comparable to those of the chloroquine-resistant strain W2, the activities of cycleanine, isochondodendrine, and 2′-norcocsuline are significantly lower in Dd2 than that reported in W2, and certainly lower than that in the chloroquine-sensitive strain D6. The semisynthetic products 4 and 5 are relatively more potent than compound 1 to 3 in Dd2, with the most potent, compound 4, being some 25.2-fold more potent than its natural precursor, cycleanine (compound 1).

**TABLE 1:** Cytotoxicity data for BBIQ alkaloids

BBIQ alkaloid (compound no.)	IC_50_ (μM) against P. falciparum strain^*a*^^,^^*d*^:			CC_50_ (μM) against cancer cell line(s):		SI^*f*^	
	Dd2	W2	D6	KB^*b*^ or HCT^*c*^	HOE^*e*^	KB/W2	HOE/Dd2
Cycleanine (1)	17.7 ± 2.0	0.25^*b*^; 4.5^*c*^	0.07^*b*^	>33.7^*b*^; 531 (HCT)^*c*^	35.0 ± 0.1	>133	2.0
Isochondodendrine (2)	6.1 ± 1.3	0.2^*c*^	ND^*d*^	29 (HCT)^*c*^	10.5 ± 1.2	116	1.7
2′-Norcocsuline (3)	7.0 ± 1.6	0.28^*b*^	0.048^*b*^	3.8^*b*^	8.0 ± 0.2	14	1.1
5-[(Dimethylamino)methyl]cycleanine (4)	0.7 ± 0.1	ND	ND	ND	10.0 ± 0.2	ND	14.3
5-[(Propargylamino)methyl]cycleanine (5)	1.8 ± 0.2	ND	ND	ND	32.0 ± 1.6	ND	17.8
Chloroquine	0.18 ± 0.03	0.135^*b*^	0.006^*b*^	33.7^*b*^	ND	250	ND

a *In vitro* 50% inhibitory concentration (IC_50_) values of BBIQ alkaloids (compounds 1 to 5) against P. falciparum chloroquine-resistant strains (Dd2 and W2 strains) and the chloroquine-sensitive strain (D6). IC_50_ values are expressed as mean ± SD for *n* = 3 independent biological repeats.

b IC_50_ data against P. falciparum W2 and D6 strains and 50% cytotoxic concentration (CC_50_) values for human oral epidermoid carcinoma (KB) cells were sourced from a previous report (16).

c IC_50_ data against chloroquine-resistant P. falciparum strain W2 and CC_50_ values for HCT-116 human colon carcinoma cells were sourced from a previous report (18).

d ND, not determined.

e CC_50_ data for human ovarian epithelial (HOE) cells. Data in this column for compounds 1 to 5 were sourced from our previous reports (13, 15).

f This selectivity index (SI) was calculated as CC_50_ /IC_50_ against P. falciparum.

Data from cytotoxicity studies of BBIQ alkaloids 1 to 3 in human oral epidermoid carcinoma (KB) or HCT-116 human colon carcinoma cells suggest low to moderate selectivity, with a selectivity index (SI) of 14 to >133. Fifty percent cytotoxic concentration (CC_50_) data for all five compounds are available from human ovarian epithelial (HOE) cells (Table 1). These data reinforce the findings of low selectivity, albeit improved in semisynthetic products 4 and 5.

### *In vivo* antimalarial activity of cycleanine (compound 1).

The isolation of the abundant cycleanine (compound 1) in *T. subcordata* root enabled us to investigate its toxicity in healthy mice and efficacy in murine malaria models after infection with Plasmodium berghei. The acute median lethal dose (LD_50_) of cycleanine after 24-h oral administration was determined to be 4.5 g/kg in mice, indicating a good safety profile. The malaria-suppressive activity of cycleanine using two oral doses (25 and 50 mg/kg of body weight/day) following P. berghei infection was demonstrated through a significant suppression of parasitemia and an increased mean survival time (MST) compared to those of untreated controls (Table 2). In particular, the higher dose (50 mg/kg/day) showed efficacy, both in terms of suppression of parasitemia and in MST, comparable to that for chloroquine at a dose of 5 mg/kg/day. The prophylactic activity of cycleanine, with the same 25 and 50 mg/kg dosing regimen during P. berghei infection in mice, was also demonstrated (Table 3). At the higher dose (50 mg/kg), cycleanine showed a suppression of parasitemia by 59.0%, only slightly less than that of 76.2% using the prophylactic pyrimethamine control at a dose of 1.2 mg/kg /day.

**TABLE 2:** Suppressive activity of cycleanine during early Plasmodium berghei infection of mice

Treatment	Dose (mg/kg) per day	Parasitemia after infection for 96 h (%)^*a*^	Suppression of parasitemia at 96 h (%)^*a*^	MST (days)^*a*^
Untreated control		28.3 ± 1.8		12.5 ± 0.3
Cycleanine	25	15.7 ± 1.8^*b*^	44.7	24.7 ± 1.1^*b*^
	50	3.8 ± 0.7^*b*^	86.5	28.2 ± 0.9^*b*^
Chloroquine	5	2.0 ± 0.8^*b*^	94.0	30.0 ± 0.0^*b*^

a Values are expressed as mean ± standard error of the mean (SEM) (*n* = 6 in each group).

b Significant relative to untreated control; *P* < 0.001.

**TABLE 3:** Prophylactic activity of cycleanine in Plasmodium berghei infection of mice

Treatment	Dose (mg/kg) per day	Parasitemia level after infection for 72 h (%)^*a*^	Suppression of parasitemia level after infection for 72 h (%)^*a*^	MST (days)^*a*^
Untreated control		20.3 ± 0.8		12.7 ± 0.3
Cycleanine	25	11.5 ± 0.9^*b*^	43.4	23.0 ± 0.6^*b*^
	50	8.3 ± 1.0^*b*^	59.0	24.5 ± 0.6^*b*^
Pyrimethamine	1.2	4.8 ± 1.1^*b*^	76.2	29.8 ± 0.2^*b*^

a Values are expressed as mean ± SEM (*n* = 6 in each group).

b Significant relative to control; *P* < 0.001.

**FIG 2:**
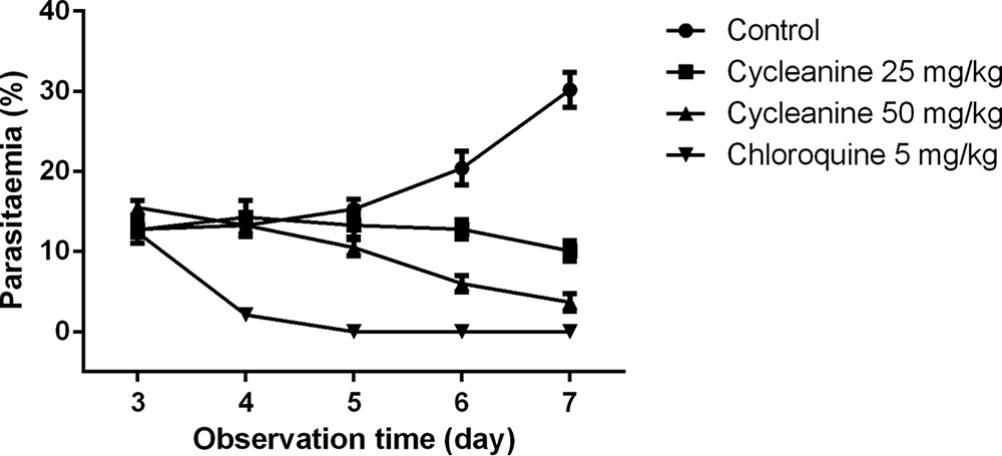
The curative activity of mice treated with cycleanine (compound 1) during established P. berghei infection. After infection of mice for 3 days, cycleanine was administered at both doses of 25 and 50 mg/kg, while water and chloroquine at 5 mg/ml were administered as negative and positive controls, respectively. The parasitemia levels were monitored for a total duration of 4 days (from day 3 to day 7).

The curative activity and MST of mice after initial P. berghei infection and subsequent treatment with cycleanine (compound 1) were determined. After infection of mice for 3 days, cycleanine was administered at both doses of 25 and 50 mg/kg and mice showed decreasing parasitemia in a dose-dependent and time-dependent manner from day 3 to day 7 (Fig. 2). The speed of killing of P. berghei parasites by chloroquine was much faster than that with cycleanine. Chloroquine reached 0% parasitemia after 5 days, while at that time cycleanine at doses of 25 and 50 mg/kg had remaining levels of 13.3 and 10.5%, respectively (Fig. 2). In this curative model, the MST of mice at doses of 25 and 50 mg/kg were 21 and 25 days, respectively, which were significantly longer than that of the control (12 days). However, they were both shorter than that of mice treated with chloroquine (30 days) (see Table S1 in the supplemental material).

### *In vivo* metabolism of cycleanine.

**FIG 3:**
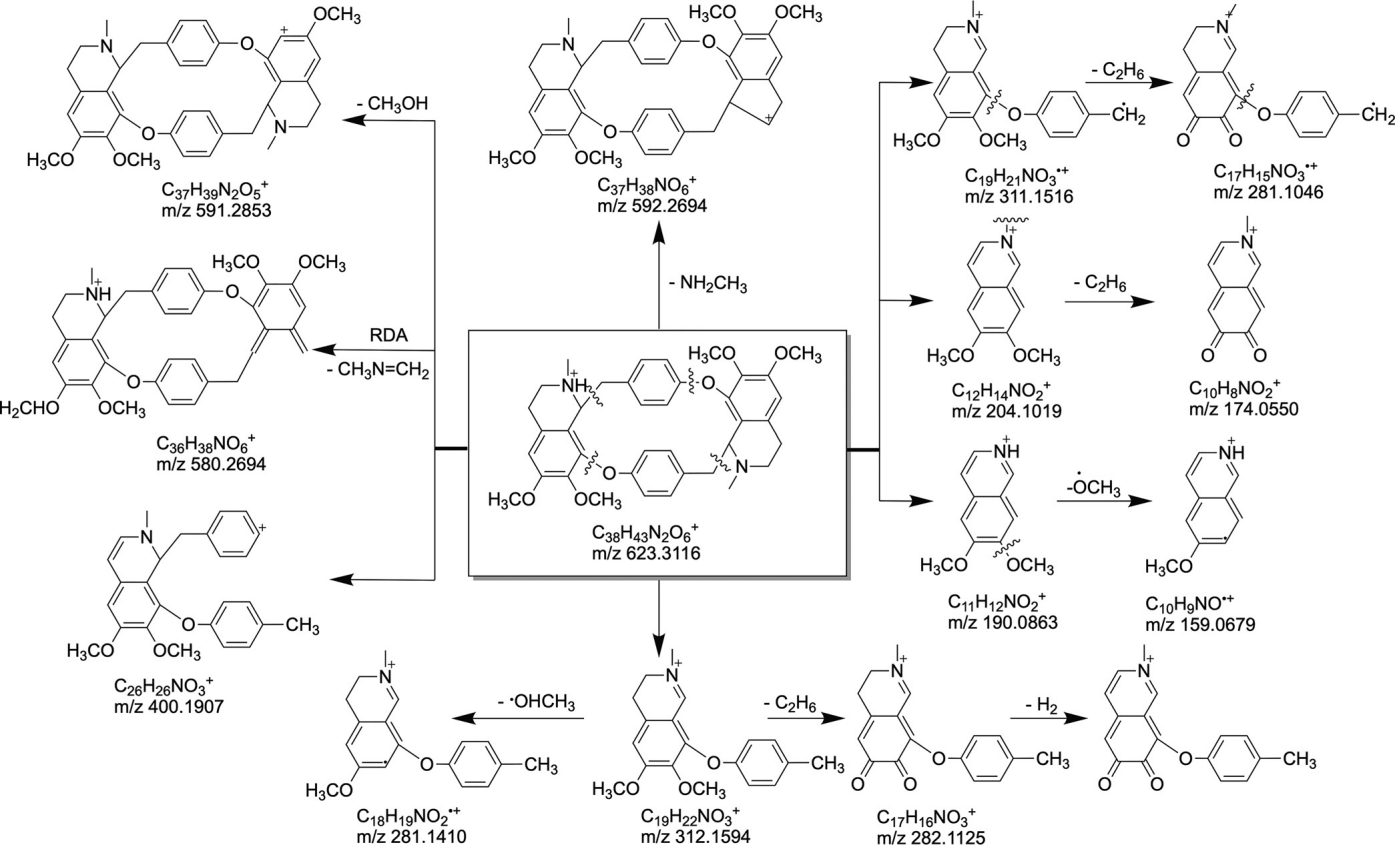
Possible fragmentation pattern of cycleanine. For analysis of fragment ions, see the text.

In order to explore the *in vivo* metabolism of cycleanine, the plasma and urine of Wistar rats following an oral dose of 120 mg/kg body weight/day over a 24-h period were analyzed for cycleanine metabolites. Samples from urine and plasma were prepared and submitted to high-performance liquid chromatography–electrospray ionization–tandem mass spectrometry (HPLC-ESI-MS/MS) analysis. The peak at the retention time of 9.7 min was cycleanine (M0) with the protonated molecular ion *m/z* of 623.3116 [M+H]^+^ (elemental composition C_18_H_43_N_2_O_6_) in the positive ion mode spectrum (Table 4 and Fig. 3; see also Fig. S2 in the supplemental material). In MS/MS, the quasimolecular ion loses a neutral molecular NH_2_CH_3_ fragment to generate an ion *m/z* of 592.2694 also by symmetric cleavage, and breaking C-O and C-C bonds to produce a fragment ion *m/z* of 312.1594, which can also lose C_2_H_6_ to produce a fragment ion *m/z* of 282.1125. After another C-O and C-C bond cleavage and the subsequent loss of CH_3_ and OCH_3_, fragment ions *m/z* 204.101, 190.0863, and 159.0679 were generated. A fragment ion *m/z* of 400.1907 was also generated by simultaneous C-O bond cleavage and C-C bond cleavage adjacent to the N atom (Fig. 3 and Fig. S2).

**TABLE 4:** HPLC-QTOF-MS retention times and mass spectrometric data of cycleanine and its metabolites

Prototype or metabolite	*t* (min)	Measured [M+H]^+^ *m/z*	Δppm	Formula	MS/MS fragment(s)	Metabolic pathway(s)	Plasma	Urine
M0	9.9	623.3125	1.41	C_38_H_43_N_2_O_6_	592.2696, 400.1895, 312.1583, 311.1508, 281.1165, 204.1011, 190.0857, 174.0911, 159.1038	Parent	+	+
M1	7.2	639.3075	1.36	C_38_H_43_N_2_O_7_	592.2472, 416.1838, 310.1422, 220.0964, 204.1046, 190.0815, 175.0955, 157.0901	Hydroxylation	−	+
M2	7.9	639.3084	2.79	C_38_H_43_N_2_O_7_	621.2977, 416.1864, 400.1917, 327.1469, 312.1361, 220.0964, 206.0780, 175.0988	Hydroxylation	+	+
M3	8.1	625.2911	0.84	C_37_H_41_N_2_O_7_	607.2784, 425.1379, 312.1591, 298.1434, 204.0999, 190.0854, 176.0691, 159.1033	Demethylation and hydroxylation	−	+
M4	9.6	609.2956	0.96	C_37_H_41_N_2_O_6_	593.2750, 427.1577, 357.1449, 312.1580, 298.1435, 204.1020, 190.0850, 176.0704, 145.0880	Demethylation	−	+
M5	10.1	595.2799	0.73	C_36_H_39_N_2_O_6_	578.2505, 284.1282, 176.0703, 145.0879	Didemethylation	−	+
M6	10.4	637.2918	1.12	C_38_H_41_N_2_O_7_	328.1553, 309.1381, 202.0855, 188.0656, 157.0879	Dehydrogenation and hydroxylation	+	+
M7	11.1	653.2855	0.38	C_38_H_41_N_2_O_8_	635.2754, 326.1384, 309.1381, 202.0855, 188.0656, 157.0879	Dehydrogenation and dihydroxylation	+	−
M8	12.1	653.2868	1.23	C_38_H_41_N_2_O_8_	592.2459, 310.1420, 293.1154, 281.1163, 269.1169, 204.1031, 190.0884	Dehydrogenation and dihydroxylation	−	+
M9	13	653.2856	0.27	C_38_H_41_N_2_O_8_	635.2701, 400.1881, 326.1380, 310.1427, 202.0855, 173.0820, 157.0881	Dehydrogenation and dihydroxylation	−	+
M10	13.4	621.2966	0.97	C_38_H_41_N_2_O_6_	591.2467, 400.1893, 398.1739, 312.1572, 310.1435, 204.1013, 202.0860, 190.0863, 188.0725, 159.1028, 157.0883	Dehydrogenation	+	−
M11	13.6	653.2859	0.73	C_38_H_41_N_2_O_8_	413.1375, 324.1595, 309.1345, 281.1158, 204.1015, 159.1021	Dehydrogenation and dihydroxylation	+	+
M12	14.1	637.2919	1.61	C_38_H_41_N_2_O_7_	594.2486, 414.1684, 326.1381, 312.1237, 281.1159, 218.0824, 204.1013, 190.0874, 173.0830	Dehydrogenation and hydroxylation	+	+

Twelve peaks on LC-MS/MS chromatograms relevant to cycleanine were detected in either urine or plasma samples (Table 4 and Fig. S3 in the supplemental material). The original form of cycleanine and 11 metabolites were found from the urine of rats, which were presumed to be hydroxylation (M1 and M2), demethylation and hydroxylation (M3), monodemethylation (M4), didemethylation (M5), dehydrogenation and hydroxylation (M6 and M12), and dehydrogenation and dihydroxylation (M7) metabolites, and the isomeric metabolites of M7 (M8, M9, and M11). From the cycleanine-containing plasma of rats, the original form cycleanine (M0) and five metabolites were found, which were presumed to be hydroxylation (M2 and M10), dehydrogenation and hydroxylation (M6 and M12), and dehydrogenation and dihydroxylation (M7) metabolites. Among them, the prototype (M0), hydroxylation (M1), and dehydrogenation and hydroxylation (M6, M12) metabolites were detected in both rat urine and plasma (Table 4 and supplemental material). Therefore, the metabolic pathway of cycleanine in rats involves hydroxylation, dehydrogenation, and demethylation or their combination, which are the main means of biotransformation of cycleanine to generate a large number of metabolites (M1 to M12) (see Fig. S5 in the supplemental material).

## DISCUSSION

Natural products (e.g., artemisinin and quinine) have demonstrated their potential as a source of antimalarial drugs. Previously, a number of BBIQ alkaloids were demonstrated to have *in vitro* antiplasmodial activities (16). Cycleanine had antiplasmodial effects with an IC_50_ of 70 nM (16) (or 80 nM [17]) against P. falciparum chloroquine-sensitive clone D6 (or 3D7) and an IC_50_ of 4.5 μM against a chloroquine-resistant strain (18). Isochondodendrine showed a low IC_50_ of 0.2 μM against a chloroquine-resistant strain (18, 19). 2′-Norcocsuline also showed potent *in vitro* antiplasmodial activity, with IC_50_ values of 48 and 248 nM against chloroquine-sensitive clone D6 (3D7) and chloroquine-resistant clone W2 (16, 20), respectively (Table 1). Our results against P. falciparum chloroquine-resistant strain Dd2 also confirmed the *in vitro* antimalarial activity of these compounds but with slightly higher IC_50_ values (Table 1) compared to the corresponding values reported in literature. Isochododendrine is a structurally demethylated analogue of cycleanine, and showed a greater potency than cycleanine in the chloroquine-resistant W2 strain and the Dd2 strain in this study (Table 1). This indicated that the increase of the hydrophilicity of cycleanine could improve its antiplasmodial activity. The SI values of all three BBIQ alkaloids ranged from 14 to 133 based on the KB or HTC-116 cells and the W2 strain, which were much greater than those based on HOE cells and the Dd2 strain. The discrepancy might be due to the different methodologies (16) used to determine IC_50_ or the different mammalian cancer cells or P. falciparum clones used. The semisynthetic analogues of cycleanine (compounds 4 and 5) produced by chemical modification of cycleanine through introduction of dimethylamino and (mono)alkynylamino groups, respectively, at the C-5 position exhibited increase in antiplasmodial potency and SI relative to cycleanine. The presence of a dimethylamino group in compound 4 could also increase the water solubility of the parent compound as is often found in the modification of other natural products, such as camptothecin (21) and thymoquinone (22). Compound 5, with a unique aminoalkynyl group, was used as a chemical probe for exploring the mechanism of action (e.g., cellular uptake) of cycleanine in cancer cells using click chemistry (15), and can also be utilized for identification of the molecular target of cycleanine in parasite-infected blood cells using a chemoproteomic approach (23). By changing the amino substitution groups, additional analogues of cycleanine with a variety of diverse structures can be synthesized for *in vitro* antiplasmodial evaluation.

To further confirm and validate the efficacy of cycleanine (compound 1) *in vivo*, its safety in healthy mice and efficacy in a murine malaria model was investigated. The LD_50_ (4.5 g/kg) of cycleanine indicated that cycleanine has a good safety profile, in agreement with the LD_50_ of 1.1 g/kg found previously in mice (24). Using suppression, prophylactic, and curative murine malaria models after infection with P. berghei (25), cycleanine showed a similar or closer effect at an oral dose of 50 mg/kg to their positive controls (chloroquine [5 mg/kg] and pyrimethamine [1.2 mg/kg]). At least, a much higher dose of cycleanine was needed to achieve the effects of these positive controls, indicating a mild efficacy *in vivo*. However, its low toxicity profile could allow increase of the oral dose (e.g., 100 mg/kg/day), which is expected to improve its efficacy. In the curative model, the slower effect of cycleanine comparing to chloroquine might be due to the metabolism of cycleanine to various metabolites. The *in vivo* antimalarial activity of cycleanine was consistent with its *in vitro* antiplasmodial activity. To our knowledge, this is the first demonstration of the *in vivo* antimalarial efficacy of a BBIQ alkaloid, cycleanine. Overall, three alkaloids (compounds 1 to 3) of *T. subcordata* could contribute to the antimalarial effects of this medicinal plant used in Nigeria for the treatment of malaria. BBIQ alkaloids of Triclisia gilletii (De Wild) Staner were also reported to be attributed to the *in vitro* and *in vivo* antimalarial activity of its plant extract (26).

Study on the metabolism of drugs can help to further understand their pharmacokinetics, efficacy, and safety (27). For example, metabolites of piperaquine were shown to have stronger antiplasmodial activity (28). However, there have been only a few *in vivo* metabolism studies of BBIQ alkaloids. Previously, *in vitro* metabolites of a BBIQ alkaloid, isoliensinine, from dog hepatic microsomes were identified as 2′-*N*-desmethylisoliensinine, 2-*N*-desmethyl-isoliensinine, and 2′-*N*-6-*O*-didesmethylisoliensinine (29). The study of the pharmacokinetics and metabolism of another BBIQ alkaloid, neferine, indicated that it was partially converted to liensinine, desmethyl-liensinine, isoliensinine, and desmethyl-isoliensinine by CYP2D6 (30). Tetrandrine was found to be initially biotransformed to a quinone methide-derived metabolite mediated by CYP3A enzymes, which was then trapped by a glutathione molecule to form a glutathione conjugate in mice (31). Metabolism of isotetrandrine *in vitro* by the rat hepatic system produced a major metabolite, *N*-desmethyl isotetrandrine (16%), and three minor oxidized metabolites, oxo-isotetrandrine (7%), hydroxy-isotetrandrine (6%), and oxohydroxy-isotetrandrine (7%), via *N*-demethylation and isoquinoline ring oxidation (32).

Our identification of 12 new metabolites of cycleanine in both plasma and urine in rats using LC-MS/MS has indicated that there are various metabolic pathways of cycleanine. These metabolites of cycleanine found in rats are also likely generated in mice after the same route of oral administration, and therefore they could contribute to its *in vivo* antimalarial efficacy found in the murine malarial model and its toxicity finding in healthy mice. Hydroxylation and demethylation of cycleanine were the common pathways consistent with those found in isoliensinine, neferine, and isotetrandrine described above. Preparation of these metabolites through chemical synthesis (33) or *in vitro* biotransformation using hepatic microsomes and/or P450 enzymes (34, 35) is possible and necessary to evaluate their potency and toxicity. Such information can be used to further guide the chemical design and modification of cycleanine to improve its potency and pharmacokinetics and to increase metabolic stability (36). Further work is necessary and ongoing in our laboratory to determine the *in vivo* antimalarial effects of BBIQ alkaloids (compounds 2 and 3) and semisynthetic derivatives (compounds 4 and 5) and the *in vitro* and/or *in vivo* antimalarial activity of the metabolites of cycleanine. Novel active drugs, particularly those with a wide safety margin, are required to help alleviate malaria morbidity and mortality and to contribute to the global control of malaria and infectious diseases.

## MATERIALS AND METHODS

### Chemicals.

Chloroquine and pyrimethamine were sourced from Sigma-Aldrich. Cycleanine (compound 1) (13), and two minor alkaloids, isochondodendrine (compound 2) and 2′-norcocsuline (compound 3), were isolated from *Triclisia subcordata* (14). Compounds 4 and 5 (Fig. 1) were previously prepared from cycleanine (compound 1) (15).

### *In vitro* antiplasmodial activity.

The evaluation of *in vitro* antiplasmodial activity of the alkaloids (compounds 1 to 3) and semisynthetic analogues (compounds 4 and 5) were performed on the intraerythrocytic P. falciparum Dd2 strain (chloroquine-resistant strain) using a SYBR green1 fluorescence dye assay as described (22, 37, 38). Compounds 1 to 5 were prepared in dimethyl sulfoxide (DMSO) with no greater than 1% of the total solvent concentration in any assay. Normalized fluorescence signals were measured against controls with 1% DMSO (100% growth) and after exposure to a supralethal concentration (10 μM) of chloroquine (0% growth). Determination of the 50% inhibitory concentration (IC_50_) was performed from a log concentration versus the mean normalized fluorescence signal curve using GraphPad Prism software (v5.0). Each biological replicate consisted of three technical repeats, with three independent biological replicates performed.

### Evaluation of the *in vivo* antimalarial activity of cycleanine.

**(i) Malaria parasite.** Chloroquine-sensitive strains of P. berghei were sourced from the National Institute of Medical Research (NIMER), Yaba Lagos, Nigeria, and maintained by subpassage in mice.

**(ii) Parasite inoculation.** Each mouse was inoculated intraperitoneally with about 1 × 10^7^ P. berghei parasitized erythrocytes in 0.2 ml of infected blood (5 × 10^7^ P. berghei erythrocytes/ml) according to published procedure (39).

**(iii) Experimental animals.** Female and male Swiss albino mice (18 to 25 g) were obtained from the University of Uyo’s animal house. Before use, mice were kept in cages and acclimatized for 10 days. All mice were kept in cross-ventilated rooms at room temperature. The care and use of mice were performed in accordance with the National Institutes of Health Guide for the Care and Use of Laboratory Animals (1996). This investigation was approved by the University of Uyo’s Animal Ethics Committee.

**(iv) Determination of median lethal dose (LD_50_) of cycleanine.** The median lethal dose (LD_50_) of cycleanine was determined using albino mice by the intraperitoneal (i.p.) route (40). Different doses of cycleanine (10 to 5,000 mg/kg) were intraperitoneally administered to groups of three mice each. The mice were monitored for manifestation of physical signs of toxicity, including decrease of motor activity, writhing, decrease of body/limb tone, weakness, and death. The number of deaths in each group within 24 h was recorded. The LD_50_ value was calculated as the geometrical means of the minimum dose producing 100% mortality and the maximum dose producing 0%.

**(v) Drug administration.** Cycleanine, chloroquine, and pyrimethamine were prepared in water and administered orally with the aid of a stainless metallic feeding cannula.

**(vi) Suppressive activity of cycleanine.** The schizontocidal activity of cycleanine and chloroquine against early P. berghei infection in mice was measured according to an established protocol (25, 41, 42). On the first day, 20 to 24 mice were infected with the parasite and randomly separated into four groups. The mice in groups 1 and 2 were given 25 and 50 mg/kg of cycleanine, respectively, those in group 3 were given 5 mg/kg of chloroquine (positive control), and those in group 4 were given distilled water (10 ml/kg, negative control) for four consecutive days. Thin films were made from the tail blood on the fifth day. Parasitized erythrocytes were counted in stained films (Giemsa stain) under a microscope. The average suppression of parasitemia (%) was calculated as follows:

$$\frac{\text{average \% parasitemia positive control}-\text{average \% parasitemia negative control}}{\text{average \% parasitemia negative control}}\times 100$$

The MST (in days) of the mice in each group was determined over a period of 30 days.

**(vii) Prophylactic activity of cycleanine.** The prophylactic activity of cycleanine was evaluated using a previously described method (42, 43). The mice were randomly divided into four groups of six mice per group. Groups 1 and 2 were given 25 and 50 mg/kg of cycleanine, respectively, group 3 was given 1.2 mg/kg of pyrimethamine (positive control), and group 4 was given 10 ml/kg of distilled water (negative control). Administration of cycleanine or the drug continued for three consecutive days. On the fourth day, the mice were inoculated with P. berghei. The parasitemia level was evaluated by blood smears after 3 days. The survival times (days) of the mice were recorded over a period of 30 days, and MST were calculated.

**(viii) Curative activity of cycleanine.** The curative activity of cycleanine was assessed according to a method described previously (42, 44). P. berghei was injected intraperitoneally into another 24 mice on the first day. Three days later, the mice were also separated into four groups of six mice per group. Groups 1 and 2 were administered different doses of cycleanine, 25 and 50 mg/kg, respectively, group 3 was given 5 mg/kg chloroquine (positive control), and group 4 was given 10 ml/kg distilled water (negative control). Cycleanine and chloroquine were given once a day for 5 days. Mouse tail blood samples were collected on each day, and Giemsa-stained thin smears were prepared to determine the parasitemia level. The MST of the mice in each group was determined over a period of 30 days.

### Metabolism of cycleanine in rats.

**(i) High-performance liquid chromatography–quadrupole time-of-flight tandem mass spectrometry.** Analysis of cycleanine metabolites was performed through high-performance liquid chromatography–quadrupole time-of-flight tandem mass spectrometry (HPLC-QTOF-MS/MS) system that consists of an Agilent 1260 HPLC coupled with an 6530 QTOF mass spectrometer with dual Agilent Jet Stream electrospray ionization source (Agilent Technologies, CA). The mass spectra were recorded in positive auto MS/MS mode, and the parameters were set as follows: temperature of drying and sheath gas, 300°C and 350°C; skimmer, 75 V; capillary voltage, 4,000 V; fragmentor, 110 V; nozzle voltage, 1,000 V; collision energy, 50 eV; pressure of nebulizer, 35 lb/in^2^; and flow rate of the drying and sheath gas, 5 and 11 liter/min, respectively. The QTOF mass spectra were recorded in high-resolution mode. The range of mass-to-charge ratio (*m/z*) scanning was set between 100 and 1,200. Samples (5 μl) were loaded onto an Agilent Poroshell 120 EC-C_18_ column (100 × 2.1 mm, 2.7 μm) at 35°C. The mobile phase consisted of water containing 0.1% formic acid (solvent A) and acetonitrile containing 0.1% formic acid (solvent B) at a flow rate of 0.35 ml/min. Gradient separation was achieved by changing the proportion of the solvent B mobile phase as follows: 0 to 2 min, 10% B; 2.1 to 5 min, 18% to 20% B; 30 to 45 min, 70% to 90% B; and 45 to 50 min, 10% B. MassHunter WorkStation software (Agilent Technologies) was utilized for the system operation and data analysis.

**(ii) *In vivo* experiments.** *In vivo* animal experiments were approved by the Animal Ethics Committee of Shanghai Institute of Materia Medica, and performed according to procedures approved by the Institutional Animal Care and Use Committee of Shanghai Institute of Materia Medica, Chinese Academy of Science. Male Wistar rats were obtained from Shanghai SLAC Laboratory Animal Co., Ltd. (Shanghai, China). The rats were given free access to water and a standard diet under controlled humidity (45% to 55%) and temperature (20°C to 24°C), except in the overnight fasting period before administration of cycleanine. The rats were adapted to the environment for a week.

Cycleanine (compound 1) was suspended in 0.4% carboxymethyl cellulose sodium (CMC-Na) and was formulated at 12 mg/ml for intragastric administration to Wistar rats (male, 220 ± 10 g, fasted for 12 h prior to administration) at a dose of 120 mg/kg body weight. Three rats were used for blood collection through the orbital vein using cannulation at 0, 0.5, 1, 2, 4, 6, 8, 12, and 24 h postdose after anaesthetization with isoflurane. The plasma samples were separated from blood by centrifugation at 12,000 rpm and 4°C for 10 min. Another three rats were placed in the metabolism cages, and urine samples were collected into tubes from 0 to 24 h after oral administration of cycleanine. All samples were stored in a −80°C freezer before analysis. Aliquots of 1.2 ml of plasma or urine samples were mixed with 3 times the volume of acetonitrile to precipitate proteins. After centrifugation at 14,000 rpm for 10 min, the supernatant was collected and evaporated under vacuum. The residue was reconstituted in 200 μl methanol, and 5 μl of each sample was injected for HPLC-QTOF-MS/MS analysis.

### Statistical analysis.

Data were expressed as mean ± standard error of the mean (SEM) for *in vivo* antimalarial experiments. Data were subjected to GraphPad Prism software analysis. Results were analyzed using one-way analysis of variance (ANOVA) followed by a *post hoc* Tukey multiple-comparison test. The difference between the mean of the experimental and control groups was considered significant at a *P* value of <0.05 (ANOVA).

### Conclusions.

Three BBIQ alkaloids of *T. subcordata*, cycleanine (compound 1), isochondodendrine (compound 2), and 2′-norcocsuline (compound 3) and two semisynthetic analogues (compounds 4 and 5) of cycleanine were demonstrated to exert significant *in vitro* antiplasmodial activities against P. falciparum. Cycleanine (compound 1) was further demonstrated to have safety and efficacy in the treatment of mice infected with P. berghei. Cycleanine was transformed to various metabolites in rats after oral delivery. The findings from this study support the use of *T. subcordata* as an antimalarial agent in traditional medicine. BBIQ alkaloids could be exploited in novel drug development in search of antimalarial agents/drugs that are urgently needed to challenge resistant plasmodium species that currently present a significant threat to human life.

## Supplementary Material

Supplemental file 1
