# Supplementary material for: In Vivo Efficacy and Metabolism of the Antimalarial Cycleanine and Improved In Vitro Antiplasmodial Activity of Semisynthetic Analogues
Source: Antimicrob Agents Chemother. 2021 Jan 20;65(2):e01995-20. doi: 10.1128/AAC.01995-20 (PMC7848973; doi:10.1128/AAC.01995-20)
Supplement: Supplemental file 1 [file AAC.01995-20-s0001.pdf]

Supplementary Materials

In vivo efficacy and metabolism of the antimalarial cycleanine and improved in vitro antiplasmodial activity of novel semisynthetic analogues

Fidelia Ijeoma Uche[1], Xiaozhen Guo[2], Jude Okokon[3], Imran Ullah[4], Paul Horrocks[4], Joshua Boateng[5], Chenggang Huang[2,*] and Wen-Wu Li[1,*]

[1] School of Pharmacy and Bioengineering, Keele University, Stoke-on-Trent, ST4 7QB, United Kingdom; fiuche@yahoo.com (FU); w.li@keele.ac.uk (WL)

[2] Shanghai Institute of Materia Medica, Chinese Academy of Science, Shanghai, 201203, China; guoxzfj@yeah.net (XG); cghuang@simm.ac.cn (CH)

[3] Department of Pharmacology and Toxicology, Faculty of Pharmacy, University of Uyo, Uyo, Nigeria; judeefiom@yahoo.com (JO)

[4] School of Medicine, Keele University, Stoke-on-Trent, ST5 5BG, United Kingdom; Imran.Ullah@utsouthwestern.edu (IU); p.d.horrocks@keele.ac.uk (PH)

[5] School of Science, University of Greenwich, Medway Campus, ME4 4TB, United Kingdom; J.S.Boateng@greenwich.ac.uk (JB)

* Correspondence: cghuang@simm.ac.cn (CH); w.li@keele.ac.uk (WL)

LC-MS/MS analysis of metabolites

The twelve metabolites (**M1-M12**) of cycleanine (**M0**) in rat were tentatively identified using LC MS/MS and described as below.

Cycleanine hydroxylation metabolite (**M1**)

The retention time of the metabolite **M1** was 7.2 min, and its protonated molecular ion was m/z 639.3056 $[M+H]^+$ (elemental composition $C_{38}H_{43}N_2O_6$) with an increase of 16 Da over M0, suggesting that it was a hydroxylated metabolite of M0. The presence of a fragment ion m/z 621.2977 suggested that one molecule H_2O was lost from its protonated molecular ion. The characteristic fragment ions m/z 206.0808, 327.1469, 416.1875, 175.0988 and 220.0964 increased 16 Da compared to the characteristic fragment ions of M0, also indicating that **M1** was a hydroxylated metabolite of cycleanine (Figure S4).

Cycleanine hydroxylation metabolite (**M2**)

The metabolite **M2** at retention time of 7.9 min gave a protonated molecular ion m/z 639.3056 $[M+H]^+$ with a 16 Da increase over M0, which was presumably another hydroxylated metabolite of M0. The fragment ion m/z 621.2963 suggested that one molecule of H_2O was lost from the molecular ion peak (Figure S4).

Cycleanine demethylation and hydroxylated metabolites (**M3**)

The retention time of the metabolite **M3** was 7.9 min, and its protonated molecular ion was m/z 625.2911 $[M+H]^+$ with 2 Da increase from **M0** (Figure S4). The fragment ion m/z of 607.2784 in the secondary mass spectrum was generated after loss of one molecule of H_2O, indicating the presence of a hydroxyl group. The ions m/z 298.1434, and 176.0691 suggested a loss of 14 Da by demethylation comparing to the corresponding fragment ions of M0, indicating that M3 is likely a demethylated and hydroxylated cycleanine metabolite.

N-demethyl cycleanine (**M4**) and didemethyl cycleanine (**M5**)

The retention times of the two metabolites **M4** and **M5** were close to 9.7 min. The protonated molecular ion of **M4** in the first-order MS was at m/z 609.2950 $[M+H]^+$, which was 14 Da less than that of **M0** (Figure S4). The fragment ions at m/z 176.0704, 145.0880, and 298.1435 were also 14 Da less than the corresponding fragment ions of M0, and other fragment ions m/z 190.085, 312.1580, 204.1020 were the same as M0 fragments, so presumably a N-demethylated metabolite. In the first order MS of M5, the quasi-molecular ion m/z 595.2799 was 28Da less than that of **M0**, whereas the fragment ion m/z 284.1282 in the MS/MS was 28 Da less than the corresponding fragment of M0. The ions m/z 578.2505, 176.0703 and 145.0879 were 14 Da less than those of M0 fragment ions. Overall, **M5** was presumably a didemethylated cycleanine metabolite.

Cycleanine dehydro and hydroxylated metabolite (**M6**)

The metabolite **M6** (Rt 10.4 min) showed the protonated molecular ion at m/z 637.2905 with 14 Da higher than that of **M0** (Figure S4). The fragment ions at m/z 157.0879, 188.0656, 202.0855, and 309.1381 in the MS/MS spectra had the characteristic fragment ions with a decrease of 2 Da, while an ion at m/z 328.1553 increased 16Da compared to the characteristic fragment of **M0**, suggesting that this metabolite may be a dehydrogenation and hydroxylation metabolite of M0, and the hydroxylation and dehydrogenation sites may be a different tetrahydrobenzylisoquinoline moiety.

Cycleanine dehydrogenation and dihydroxylated metabolites (**M7**)

The metabolite **M7** had a retention time of 11.1 min with the protonated molecular ion peak at m/z 653.2855 $[M+H]^+$ (elemental composition $C_{38}H_{41}N_2O_8$) with 30 Da more than M0 (Figure S4). Secondary fragment ions at m/z 635.2754 $[M-H_2O]^+$, 157.0879, 202.0855, 188.0656, 309.1381 were 2 Da less than M0 characteristic fragment ions. In addition, a characteristic fragment ion at m/z 326.1384s was 14 Da greater than that of M0, indicating that the dehydrogenation site and one of the

hydroxylation sites were in the same tetrahydro-benzylisoquinoline fragment, so this metabolite was presumed to be a dehydrogenated and dihydroxylated metabolite of cycleanine.

Cycleanine dehydrogenation and dihydroxylated metabolite **M7** isoforms (**M8, M9, M11**)

The retention times of the metabolites **M8, M9** and **M11** were 12.1, 13.0 and 13.6 min, respectively, and their protonated ions were all m/z 653.2868 $[M+H]^+$, and the characteristic fragment ions at m/z 312.1586, 190.0884, 204.1031 were consistent with the corresponding fragments of **M0** (Figure S4) , suggesting that they were isomeric metabolites of **M7** as the dehydrogenated and dihydroxylated cycleanine.

Cycleanine dehydrogenation metabolite (**M10**)

The retention time of metabolite **M10** was 13.6min with the protonated molecular ion at m/z 621.2966 $[M+H]^+$, decreased by 2Da comparing to M0. The fragment ions in the second-order MS m/z 157.0883, 188.0725, 202.0860, 310.1435, and 398.1739, showed similar pattern to the corresponding fragments of M0 as 159.1028, 190.0863, 204.1013, 312.1572 and 400.1893, but with a decrease of 2 Da (Figure S4). Therefore, **M10** was presumed to be a dehydrogenation metabolite of cycleanine.

Cycleanine dehydro and hydroxylated metabolite (**M12**)

The metabolite **M12** with retention time at 14.1 min gave a protonated molecular ion at m/z 637.2905 $[M+H]^+$. The fragment ions 190.0874, 204.013 and 312.1237 in the MS/MS spectra were the same as the corresponding characteristic fragments of **M0**, while fragment ions at m/z 218.0824, 326.1381, and 414.1684 increased by 14 Da compared to the corresponding characteristic fragments of **M0** (Figure S4). It was presumed that the sites of dehydrogenation and hydroxylation in this metabolite were in the same tetrahydro-benzylisoquinoline moiety.

Table S1: Curative activity and mean survival time (MST) of mice treated with cycleanine (**1**) during established *P. berghei* infection for 3 days.

Treatment	Dose (mg/kg)	Parasitaemia (%)[a]					MST (day)[a]
		Day 3	Day 4	Day 5	Day 6	Day 7	
Control	-	12.8 ± 1.0	13.3 ± 1.2	15.3 ± 1.2	20.4 ± 2.1	30.2 ± 2.2	12.2 ± 0.2
Cycleanine	25	12.7 ± 1.7	14.3 ± 2.1	13.3 ± 1.5	12.8 ± 1.2	10.1 ± 1.3	21.3 ± 0.8[b]
	50	15.5 ± 0.9	13.3 ± 1.4	10.5 ± 1.0	6.0 ± 1.0	3.7 ± 1.1	24.8 ± 0.4[b]
Chloroquine	5	12.4 ± 0.5 [a]	2.1 ± 0.2	0.0	0.0	0.0	29.8 ± 0.2[b]

[a] Values are expressed as mean ± SEM (n = 6 in each group)
[b] Significant relative to control, $p < 0.001$.

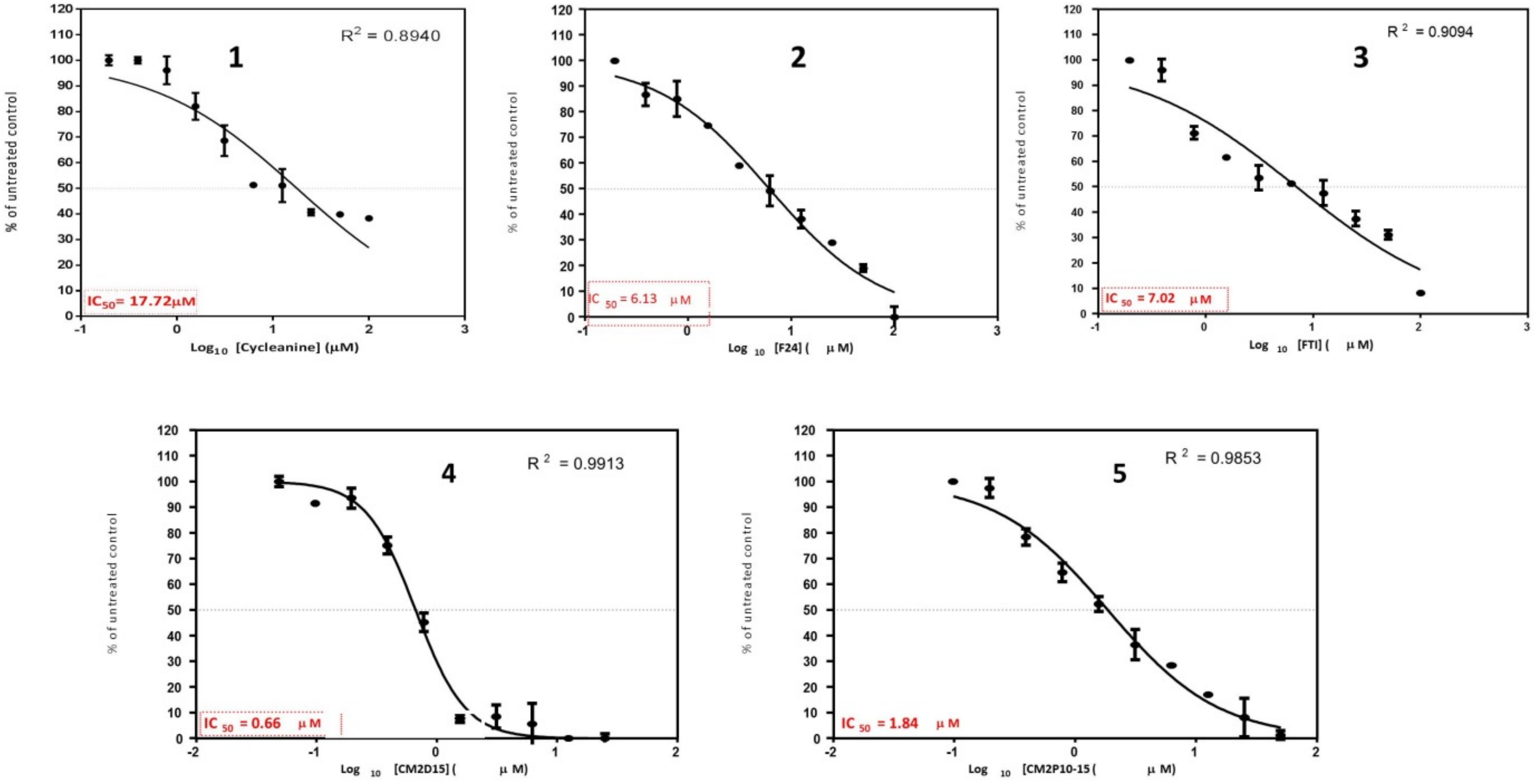

Figure S1. Dose-response anti-plasmodial curves of BBIQ alkaloids (**1-5**).

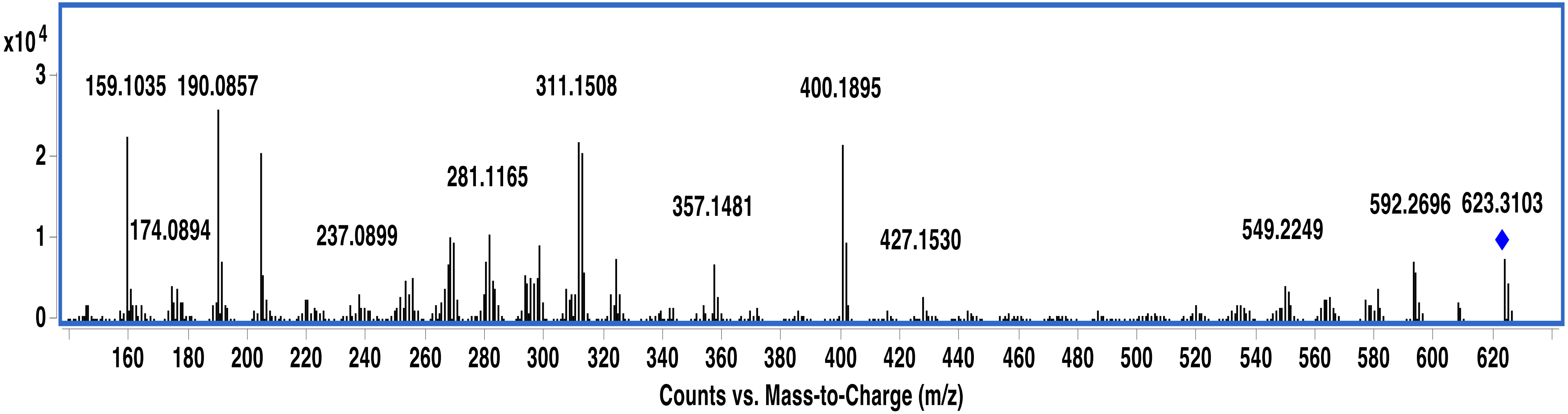

Figure S2. HPLC ESI-MS/MS spectrum of cycleanine.

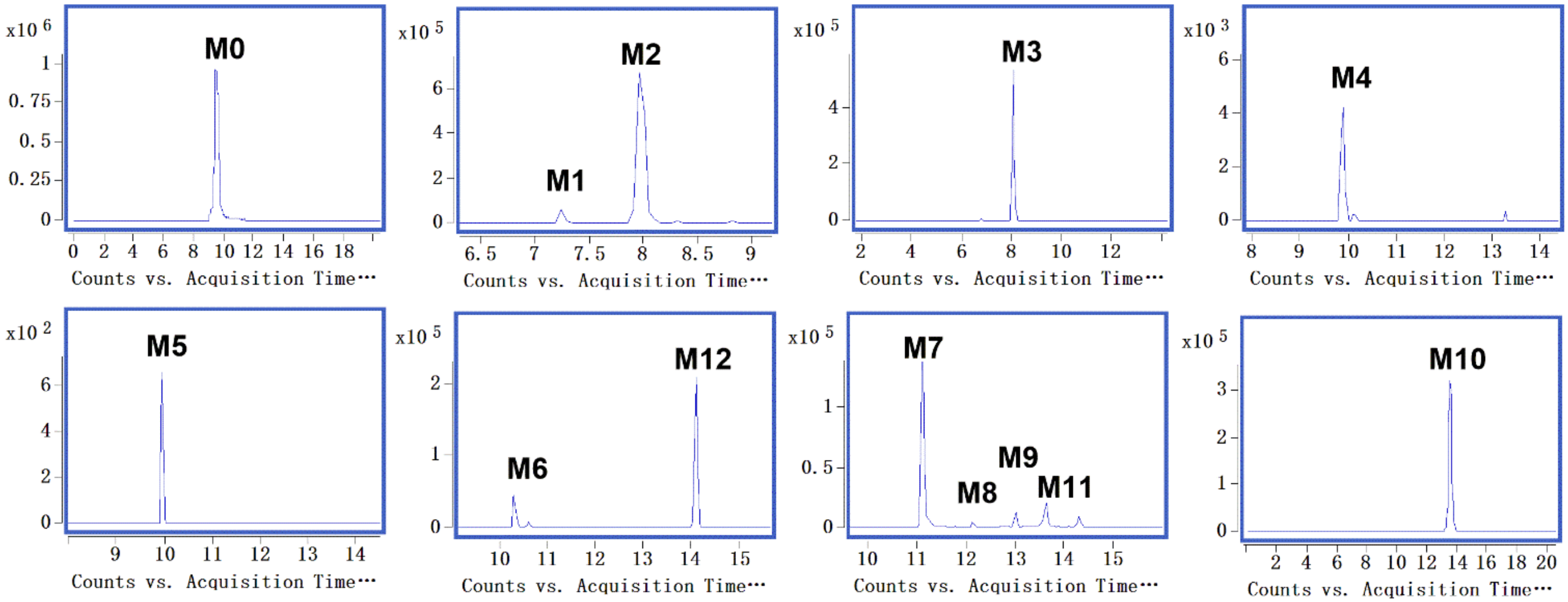

Figure S3. Extracted ion chromatograms of cycleanine (**M0**), its 5 metabolites (**M2, M6, M7, M10** and **M12**) in plasma, 11 metabolites (**M1-6, M7-9, M11-12**) in urine of rat. **M0, M1, M6**, and **M12** were present in both urine and plasma.

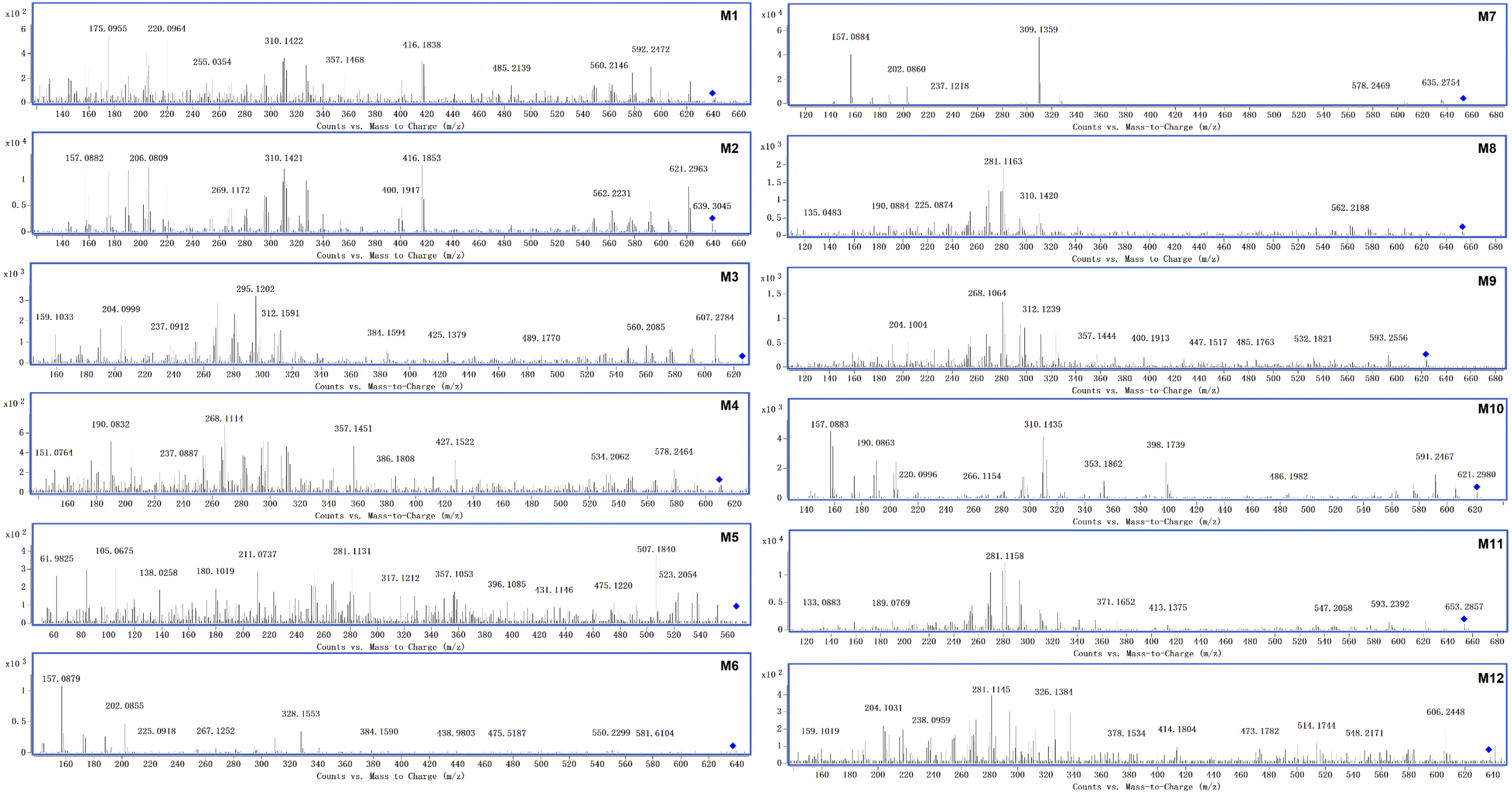

Figure S4. MS/MS spectra of cycleanine metabolites (**M1-M12**).

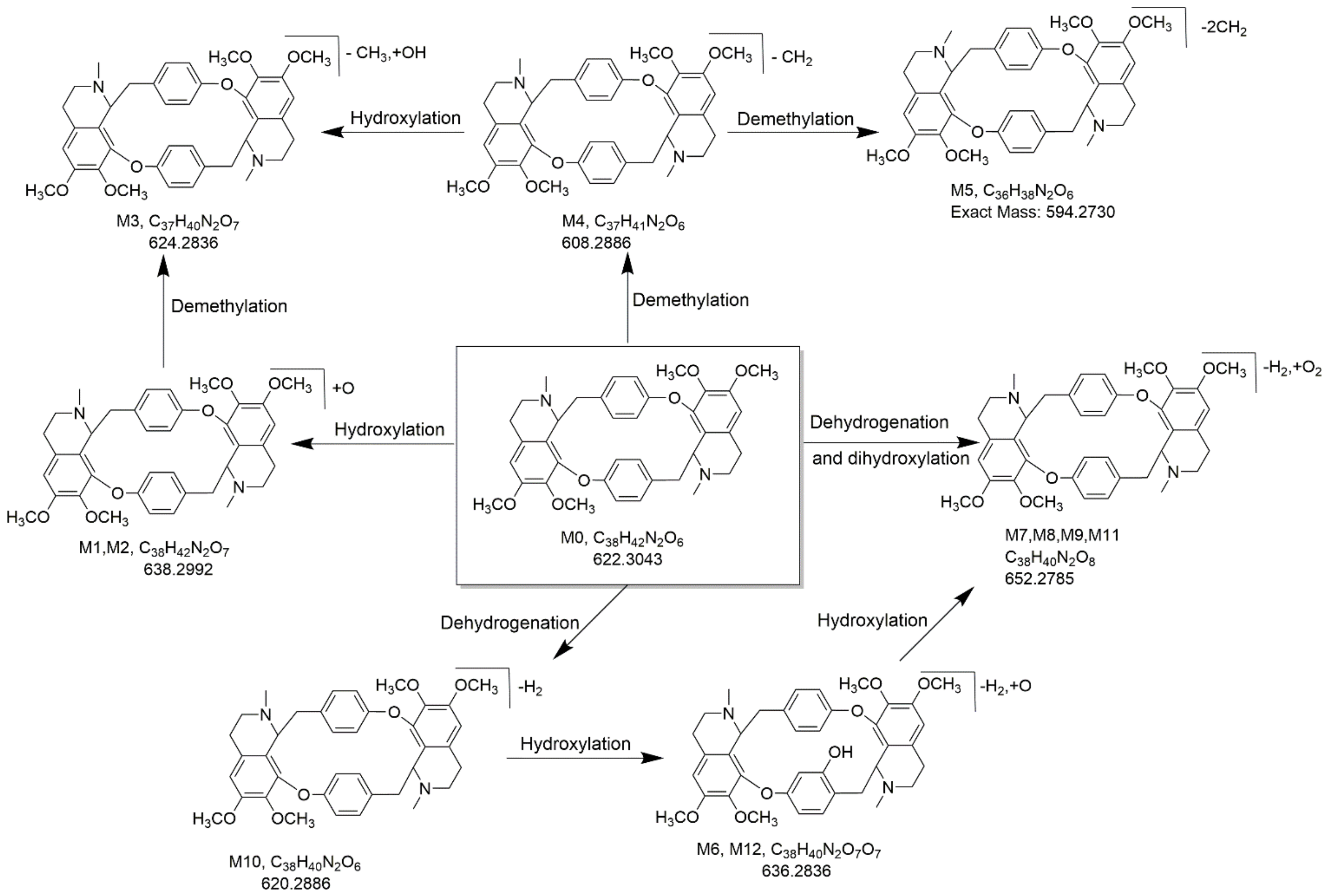

Figure S5 Possible metabolic pathway of cycleanine in rats.
